# Intermediate-term risk of cardiac allograft vasculopathy following heart transplantation from hepatitis C viremic donors in the era of direct-acting antiviral therapy

**DOI:** 10.1016/j.jhlto.2025.100416

**Published:** 2025-10-25

**Authors:** K. Paternostro, A. Birs, S. Aslam, E. Adler, K. Hong, N. Wettersten

**Affiliations:** Division of Cardiology, Department of Medicine, University of California San Diego Health, San Diego, CA

**Keywords:** Heart transplantation, Cardiac allograft vasculopathy, Hepatitis C virus, Intravascular ultrasound, Antivirals

## Abstract

**Background:**

Cardiac allograft vasculopathy (CAV) is a leading cause of death in heart transplant (HTx) recipients. Chronic hepatitis C virus (HCV) infection has been associated with increased inflammation and accelerated CAV. The advent of direct-acting antiviral (DAA) therapy has renewed interest in transplanting HCV-viremic donors, though long-term outcomes remain limited.

**Methods:**

We conducted a single-center retrospective study of adult HTx recipients at UC San Diego from 2015 to 2019 who underwent routine intravascular ultrasound (IVUS) surveillance. Recipients were stratified by donor HCV nucleic acid amplification test (NAT) status. Donor-derived HCV infection was treated with DAA therapy. We used multivariable-adjusted Cox regression to evaluate the primary endpoint of developing CAV, defined as maximal intimal thickness (MIT) ≥ 0.5 mm, and endpoints of MIT ≥ 0.7 mm and a composite outcome of incident acute coronary syndrome, percutaneous coronary intervention (PCI), and all-cause mortality.

**Results:**

Among 131 recipients, 22 received HCV NAT+ hearts. Baseline donor and recipient characteristics were similar, except for recipients of HCV NAT- hearts were younger (53.7 years vs 61.0 years; *P* = 0.022). Over a median follow-up of 6.6 years, HCV NAT+ status was not associated with a higher risk of CAV (MIT ≥ 0.5 mm: adjusted HR 0.89, 95% confidence interval (CI), 0.52-1.53; *p* = 0.673; MIT ≥ 0.7 mm: adjusted HR 0.91, 95% CI 0.51-1.61; *p* = 0.750), nor the composite outcome (adjusted HR 1.23, 95% CI, 0.45-3.40; *p* = 0.690).

**Conclusion:**

In the modern DAA era, transplantation of HCV NAT+ donor hearts is not associated with increased risk of CAV or adverse clinical outcomes over intermediate-term follow-up.

## Background

Cardiac transplantation is a life-saving procedure, with transplant rates increasing by more than 80% since 2011.^1^ However, the number of individuals awaiting transplant continues to outpace donor availability year over year. To meet the growing demand for donor hearts, efforts are ongoing to expand the donor pool to include non-traditional sources, such as hearts from donors after circulatory death (DCD), those preserved through ex-vivo perfusion, and donors previously considered high-risk, including those positive for hepatitis C virus (HCV).^2^, ^3^ Interest in utilizing HCV-viremic donor hearts has grown in parallel to the opioid epidemic in the United States, with climbing rates of injection drug use, transmission of HCV, and overdose-related deaths.^4^, ^5^ This rise in opioid-related deaths has led to an increase in the availability of suitable organs from younger donors with fewer medical comorbidities.

Resistance to utilizing HCV-viremic donors partially stems from studies showing accelerated cardiac allograft vasculopathy (CAV) and higher mortality in recipients of hearts from HCV-viremic and seropositive donors.^6^, ^7^, ^8^ However, much of this data was obtained prior to the widespread availability and utilization of direct-acting antivirals (DAA) and the associated high cure rates of HCV. Contemporary studies have demonstrated similar short-term outcomes of mortality and rejection in patients transplanted with HCV viremic hearts, who achieved sustained virologic response (SVR) with a short course of DAA therapy, compared to non-HCV donors.^9^, ^10^, ^11^ These findings have prompted renewed interest in the use of HCV-viremic donor hearts, with national data from the United Network for Organ Sharing showing a substantial increase in utilization, from just three transplants in 2015 to over 450 in 2023, reflecting a significant expansion of the donor pool. While our center, along with others, has demonstrated a similar burden of early CAV development between these populations, the follow-up period may be insufficient to assess this chronic, often late-presenting disease thoroughly. The long-term risk of CAV development associated with HCV-positive donor hearts remains uncertain and warrants further investigation.

In this study, we assessed the association between HCV donor status and incident CAV over intermediate follow-up in the contemporary era of DAA therapies. We hypothesized that risk for CAV would be comparable in donor hearts with and without HCV viremia.

## Materials and methods

### Participants

This was a single-center, retrospective cohort study of consecutive heart transplant (HTx) recipients between January 2015 and January 2019 at the University of California, San Diego. All transplant recipients with at least one surveillance coronary angiogram with intravascular ultrasound (IVUS) for CAV were included. HTx recipients either received an HCV-viremic heart, as determined by a positive nucleic amplification test (HCV NAT+), or a non-viremic (HCV NAT−) heart. Patients were included irrespective of illness severity, listing status, or whether dual organ transplantation was performed. A standardized immunosuppression regimen with tacrolimus, mycophenolate, and steroids was utilized with selective use of induction therapy (basiliximab or thymoglobulin) based on recipient risk profile for rejection. Donor information was collected via data query from the United Network for Organ Sharing database.

### HCV surveillance and treatment protocol

During the study period, our institution was among the early adopters of transplanting HCV-viremic donor hearts as part of clinical practice. At that time, a reactive approach to DAA therapy was employed. All recipients of HCV NAT+ donor hearts underwent pre-transplant HCV ELISA and polymerse chain reaction (PCR) testing. Post-transplant HCV PCR was obtained on postoperative day 3, week 1, and week 2, along with HCV genotyping, subtyping, and NS5A resistance testing if HCV PCR was positive. PCR was repeated on the day of discharge or immediately prior to initiation of antiviral therapy. Upon confirmation of viremia, patients were treated with a standardized 12-week course of DAA therapy—typically glecaprevir/pibrentasvir, sofosbuvir/velpatasvir, or ledipasvir/sofosbuvir. Following treatment initiation, HCV PCR was obtained every 4 weeks for 24 weeks from the initiation of DAA therapy, with a final HCV PCR and ELISA drawn at 12 months post-transplant. All patients achieved a SVR, defined as the absence of detectable HCV ribonucleic acid by PCR testing, within 12 weeks after completing the treatment course. In recent years, our protocol has transitioned to a prophylactic strategy with a 7-day course of glecaprevir/pibrentasvir starting within 8 hours prior to transplant surgery to prevent HCV transmission to the recipient.^12^

### IVUS assessment and data collection

Routine CAV surveillance by cardiac catheterization with IVUS began at the first annual visit and continued yearly until 5 years post-transplantation but could be shortened or lengthened by the treating transplant cardiologist. Intimal thickness was evaluated with IVUS, with measurements made by the performing interventional cardiologist (Polaris Multi-Modality Guidance System, Boston Scientific Inc., USA). IVUS data collected during annual surveillance studies were gathered using retrospective reviews (A.B., K.P., and A.K.). They included the location and centrality of the lesion, the degree of stenosis, a qualitative description of the lesion, and the minimum lumen area (MLA). IVUS was used in this study to diagnose and quantify CAV, which may be angiographically occult.^13^, ^14^

### Outcomes

Our primary outcome was the development of CAV, defined as a maximal intimal thickness (MIT) ≥ 0.5 mm at any point during the follow-up period in accordance with national standards and following the Stanford IVUS classification system.^15^

Secondary outcomes included an alternative definition of CAV, defined more restrictively as MIT ≥ 0.7 mm. This cutoff was chosen based on recent evidence suggesting that higher MIT thresholds were associated with increased risk of worse clinical outcomes.^16^ Additionally, we evaluated the association between HCV donor status and a composite endpoint comprising incident acute coronary syndrome (as defined by incident NSTEMI/STEMI), percutaneous coronary intervention (PCI), and all-cause mortality.

### Statistical analysis

Baseline characteristics between recipients of HCV NAT− and HCV NAT+ donor hearts were compared with Student’s *t*-test, Mann-Whitney *U*, and chi-square test as appropriate.

The association between HCV donor status and outcomes was assessed with univariable and multivariable Cox regression models. Covariates for adjustment in multivariable models were selected using bidirectional selection, with the final model selected based on covariates that had the lowest Akaike Information Criterion. Donor variables evaluated for inclusion were age, sex, race, body mass index (BMI), tobacco use, and history of hypertension and diabetes. Recipient variables evaluated for inclusion were age, sex, race, BMI, tobacco use, history of hypertension, diabetes, and metabolic syndrome (defined as three or more of the following: BMI > 30, low high-density lipoprotein (HDL) (<50 in women, <40 in men), triglycerides >150 mg/dL, prevalent diabetes, prevalent hypertension), ischemic or non-ischemic cardiomyopathy, creatinine at time of transplant, and low-density lipoprotein level (LDL) at time of transplant. Non-donor or recipient variables evaluated included the use of induction at the time of transplant, multiorgan transplantation, cytomegalovirus (CMV) infection risk post-transplant, abnormal AlloMap post-transplant, the number of episodes of acute cellular rejection (≥2R), as well as the average LDL, sirolimus use, and high-intensity statin use in the first year after transplant.

Final multivariable models for the primary outcome included donor age, sex, BMI, and diabetes, as well as recipient metabolic syndrome, induction medication use, average LDL in the first year after transplant, and high-intensity statin use in the first year after transplant. Variables in the final model for the secondary outcome of MIT ≥ 0.7 mm included donor age, race, diabetes, and hypertension, as well as induction medication use and average LDL in the first year after transplant. Variables in the final model for the composite outcome included creatinine at the time of transplant, CMV risk, and abnormal AlloMap after transplant. In a sensitivity analysis, we assessed the MIT outcomes using competing risks analysis to account for the competing risk of death.^17^ We investigated potential differences in the annual rate of change in MIT between recipients of HCV NAT− and HCV NAT+ donor hearts using adjusted linear mixed models. Lastly, while multiorgan transplantation was not significant in the bidirectional selection of covariates, studies have shown that multiorgan transplantation is protective for CAV; thus, we performed sensitivity analyses evaluating for effect modification by testing the interaction of HCV status with multiorgan transplant status and evaluating outcomes in individuals undergoing HTx alone.^18^

A *p*-value of <0.05 was regarded as statistically significant for all analyses. All statistical analyses were performed using (R Core Team, 2021). R: A language and environment for statistical computing. R Foundation for Statistical Computing, Vienna, Austria), version 4.2.2.

## Results

### Baseline characteristics

A total of 131 HTx recipients were included in this analysis. The average age of recipients was 55 ± 14 years, with 88% male. Forty percent self-identified as White, 24% had diabetes, and 30% had metabolic syndrome (Table 1). Most recipients had non-ischemic cardiomyopathies (63%), and 15% received multiorgan transplants. Of the 131 HTxs, 22 received hearts from HCV NAT+ donors. Compared to individuals receiving HCV NAT− hearts, those who received HCV NAT+ hearts were significantly older *(p* = 0.022), though no other significant differences were observed. There were no differences in donor characteristics between the two groups.

**Table 1:** Recipient and Donor Baseline Characteristics

Recipient characteristics	NAT negative (*N* = 109)	NAT positive (*N* = 22)	*p*-value
Age, mean (SD), years	53.7 (14)	61.0 (8)	0.022
Male, *N* (%)	95 (88)	21 (95)	0.454
Race, *N* (%)			0.406
White	44 (40.4)	8 (36.4)	
Black	8 (7.3)	4 (18.2)	
Asian/Pacific Islander	10 (9.2)	1 (4.5)	
Other	47 (43.1)	9 (40.9)	
Tobacco use, *N* (%)	53 (48.6)	9 (40.9)	0.669
BMI, mean (SD), kg/m²	26.0 (4.7)	26.5 (3.29)	0.632
Hypertension, *N* (%)	96 (88.1)	19 (86.4)	>0.999
Diabetes, *N* (%)	25 (22.9)	7 (31.8)	0.540
LDL at transplant, mean (SD)	76.1 (28.9)	78.5 (38.7)	0.737
Triglycerides at transplant, median [IQR]	109.0 [79.9, 148.0]	104.5 [87.0, 138.3]	0.707**
HDL at transplant, median [IQR]	39.0 [31.0, 48.0]	39.5 [29.5, 52]	0.803**
Metabolic syndrome, *N* (%)^a^	32 (29.4)	7 (31.8)	>0.999
Total Ischemic time, mean (SD), minutes	194.9 (49.7)	214.3 (45.0)	0.174
Multiorgan transplant, *N* (%)	17 (15.6)	2 (9.1)	0.647
Concomitant liver transplant, *N* (%)	2 (1.8)	0 (0.0)	>0.999
Concomitant kidney transplant, *N* (%)	13 (11.9)	2 (9.1)	>0.999
Concomitant lung transplant, *N* (%)	2 (1.8)	0 (0.0)	>0.999
Nonischemic cardiomyopathy, *N* (%)	71 (65.1)	12 (54.5)	0.485
HCV clearance at 12 Months, *N* (%)	-	22 (100)	-
CMV risk stratification, *N* (%)			0.154
Low risk (D−/R− or D−/R+)	38 (34.9)	4 (18.2)	
Moderate risk (D+/R+)	50 (45.9)	15 (68.2)	
High risk (D+/R-)	21 (19.3)	3 (13.6)	
Number of ACR ≥ 2 episodes, *N* (%)			0.678
Zero	82 (75.2)	18 (81.8)	
One	20 (18.3)	4 (18.2)	
Two	4 (3.7)	0 (0.0)	
Three	3 (2.8)	0 (0.0)	
Abnormal AlloSure, *N* (%)^b^	16 (34.8)	7 (77.8)	0.043
Abnormal AlloMap, *N* (%)^c^	98 (95.2)	22 (100)	0.585
Transitioned to mTORi by year 1 Post-OHT	47 (43.1)	12 (54.6)	0.355
Donor characteristics			
Age, median [IQR], years	32.0 [24.0, 41.0]	35.0 [30.0, 44.8]	0.101
Male, *N* (%)	89 (81.7)	19 (86.4)	0.824
Race, *N* (%)			0.492
White	59 (54.1)	15 (68.2)	
Black	10 (9.2)	1 (4.5)	
Asian/Pacific Islander	6 (5.5)	0 (0.0)	
Other	34 (31.2)	6 (27.3)	
Tobacco use, *N* (%)			0.124
No	99 (90.8)	17 (77.3)	
Yes	9 (8.3)	5 (22.7)	
Unknown	1 (0.9)	0 (0.0)	
Hypertension, *N* (%)			0.553
No	89 (81.7)	20 (90.9)	
Yes	19 (17.4)	2 (9.1)	
Unknown	1 (0.9)	0 (0.0)	
Diabetes, *N* (%)			0.659
No	105 (96.3)	22 (100.0)	
Yes	3 (2.8)	0 (0.0)	
Unknown	1 (0.9)	0 (0.0)	

ACR, acute cellular rejection; IQR, interquartile range; mTORI, mammalian target of rapamycin inhibitor; NAT, nucleic acid amplification test; OHT, orthotopic heart transplant; SD, standard deviation.

** Indicates non-normal distribution.

a Metabolic Syndrome is defined as greater than or equal to three of the following: Body Mass Index (BMI) > 30 kg/m^2^, Low HDL (<40 mg/dL in men, <50 mg/dL in women), Triglycerides >150 mg/dL, prevalent diabetes or prevalent hypertension.

b Abnormal AlloSure defined as any reading ≥0.15%.

c Abnormal AlloMap defined as any reading ≥30.

### Maximal intimal thickening

**Figure 1:**
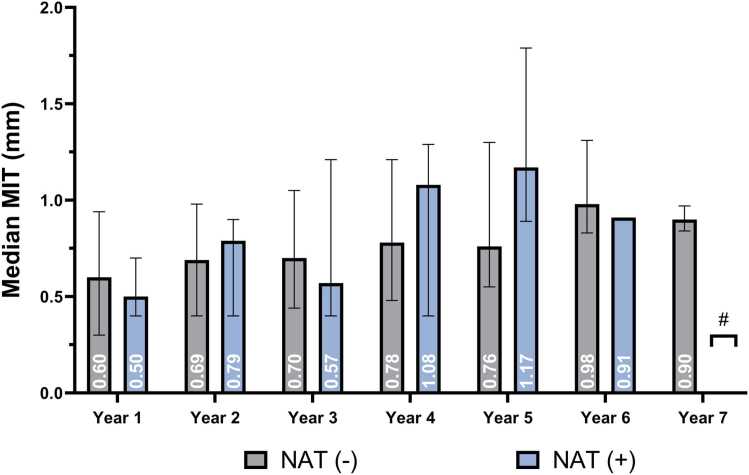
Median annual mean intimal thickness (MIT) following heart transplantation in recipients of HCV NAT− and HCV NAT+ donor hearts over 7 years of follow-up. Median MIT (in mm) is shown for each year post-transplant, with gray bars representing NAT− and blue bars representing NAT+ recipients. Error bars indicate interquartile range. # At year 7, no NAT+ recipients had available follow-up; therefore, only NAT− values are shown. No significant differences in median annual MIT were observed between groups at any time point.

There was an average of 3.5 ± 1.5 IVUS studies per recipient over a median follow-up of 6.6 years, with a maximum follow-up of 9.7 years. There was no difference in median MIT values between recipients of HCV NAT− and HCV NAT+ donor hearts each year of follow-up (Figure 1). MIT increased by 0.1 mm annually on average, and this rate of change did not significantly differ by HCV donor status in either unadjusted (p*interaction = 0.152) or adjusted linear mixed models (p*interaction = 0.147) in linear mixed models. Similarly, when evaluating the change in MIT in individuals undergoing heart transplantation alone, MIT increased by 0.1 mm on average annually, which did not significantly differ by HCV status (p*interaction = 0.136 in the adjusted model).

### Association of HCV status with CAV

**Figure 2:**
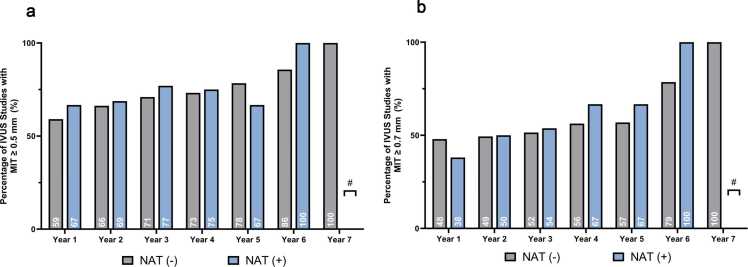
Proportion of intravascular ultrasound (IVUS) studies demonstrating significant intimal thickening in recipients of HCV NAT− and HCV NAT+ donor hearts over 7 years of follow-up. **(A)** Percentage of IVUS studies with mean intimal thickness (MIT) ≥ 0.5 mm. **(B)** Percentage of IVUS studies with MIT ≥ 0.7 mm. Gray bars represent NAT− recipients; blue bars represent NAT+ recipients. # At year 7, no NAT+ recipients had available follow-up; therefore, only NAT− values are shown. No statistically significant differences were observed between groups at any time point.

During the study period, 101 (83%) of the HTx recipients developed an MIT ≥ 0.5 mm (Figure 2). The risk of developing MIT ≥ 0.5 mm was not different in recipients of HCV NAT+ donor hearts in either unadjusted (hazard ratio [HR] 1.00, 95% confidence interval [CI], 0.58-1.87; *p* = 0.994) or adjusted analysis (HR 0.89, 95% CI, 0.52-1.53; *p* = 0.673). There was no significant interaction by multiorgan transplantation status (p*interaction = 0.323), and the risk estimate was similar in individuals undergoing heart transplantation only (HR 1.00, 95% CI 0.56-1.77). This association did not differ in competing risk analysis either (subdistribution HR [sHR] 0.90, 95% CI, 0.49-1.65; *p* = 0.740) (Table 2).

**Table 2:** Comparison of NAT Negative and NAT Positive Groups Regarding CAV Development and Secondary Outcomes

	NAT negative (*N* = 109)	NAT positive (*N* = 22)	*p*-value
Mean follow-up	6.7 years	6.1 years	
Average days from OHT to angiogram by year, mean (SD), days			
Year 1	373.4 (31.5)	392.1 (61.5)	0.239
Year 2	755.8 (79.3)	789.3 (124.8)	0.176
Year 3	1091 (119.7)	1070 (166.0)	0.550
Year 4	1441 (145.5)	1424 (204.9)	0.600
Year 5	1764 (146.5)	1721 (258.8)	0.836
Year 6	1994 (266.0)	1553 (384.7)	0.121
Year 7	2288 (528.2)	1505 (N/A)	-
Development of CAV (0.5 mm)			
No. of events	85/109	16/22	
Unadjusted hazard ratio (95% CI)	1.00 (0.58, 1.70)		0.994
Adjusted hazard ratio (95% CI)^a^	0.89 (0.52, 1.53)		0.673
Sub distribution hazard ratio (95% CI)	0.90 (0.49, 1.65)		0.740
Development of CAV (0.7 mm)			
No. of events	69/109	15/22	
Unadjusted hazard ratio (95% CI)	1.04 (0.59, 1.81)		0.905
Adjusted hazard ratio (95% CI)^b^	0.91 (0.51, 1.61)		0.750
Sub distribution hazard ratio (95% CI)	0.93 (0.56, 1.53)		0.760
Secondary outcomes			
No. of events (total)	27	9	
Acute coronary syndrome	6	2	
Percutaneous intervention	11	4	
All-cause mortality	10	3	
Unadjusted hazard ratio (95% CI)	1.35 (0.51, 3.60)		0.549
Adjusted hazard ratio (95% CI)^c^	1.23 (0.45, 3.40)		0.690
Sub distribution hazard ratio (95% CI)	0.93 (0.56, 1.53)		0.760

CAV, cardiac allograft vasculopathy; NAT, nucleic acid amplification test; OHT, orthotopic heart transplant; SD, standard deviation.

a Adjusted for Donor Age, Sex, BMI, and prevalence of diabetes, average LDL one-year post-transplantation, presence of a high intensity statin, prevalent metabolic syndrome in recipient, induction medication.

b Adjusted for Donor Age, Race, and prevalence of diabetes and hypertension, average LDL one-year post-transplantation, induction medication.

c Adjusted for Recipient creatinine at transplantation, CMV risk stratification, and abnormal Allomap at any time post-transplantation.

Eighty-four (64%) of the HTx recipients developed an MIT ≥ 0.7 mm. HCV NAT donor status again was not associated with greater risk of CAV in either unadjusted (HR 1.04, 95% CI, 0.59-1.81; *p* = 0.905) or adjusted analysis (HR 0.91, 95% CI 0.51-1.61; *p* = 0.750). There was no significant interaction by multiorgan transplantation status (p*interaction = 0.876), with a similar risk estimate in individuals undergoing heart transplantation only (HR 1.01, 95% CI 0.55-1.84). Associations were similar in competing risks analysis (sHR 0.93, 95% CI, 0.56-1.53; *p* = 0.760).

### Association of HCV status with composite outcome

**Figure 3:**
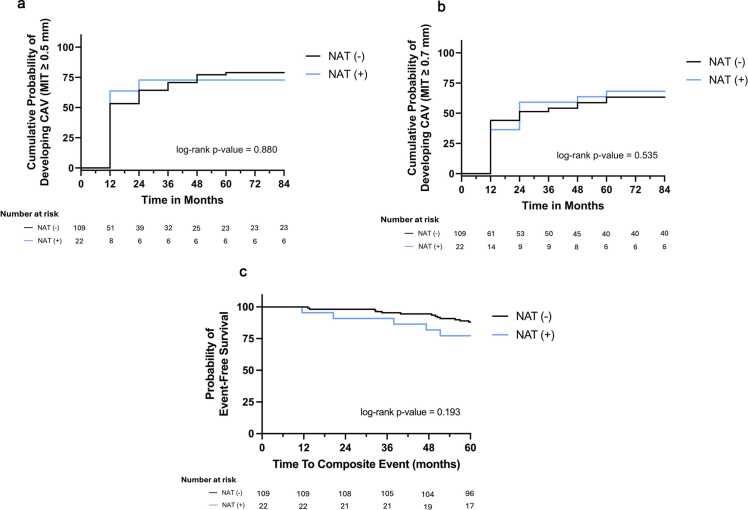
Kaplan-Meier curves comparing outcomes in recipients of HCV NAT− and HCV NAT+ donor hearts, with the number at risk listed below each graph. **(A)** Cumulative probability of developing cardiac allograft vasculopathy (CAV), defined as mean intimal thickness (MIT) ≥ 0.5 mm. **(B)** Cumulative probability of developing CAV with a more restrictive threshold of MIT ≥ 0.7 mm. **(C)** Event-free survival for the composite outcome of acute coronary syndrome (NSTEMI or STEMI), percutaneous coronary intervention (PCI), or all-cause mortality. Gray lines represent NAT− recipients; blue lines represent NAT+ recipients. Log-rank *p*-values: 0.880 **(A)**, 0.535 **(B)**, and 0.193 **(C)**. No statistically significant differences were observed.

Twenty-five (19%) individuals experienced the composite outcome over a maximum follow-up of 9.7 years. HCV NAT+ donor status was not associated with a greater risk of the composite outcome in either unadjusted (HR 1.35, 95% CI, 0.51-3.60; *p* = 0.549) or adjusted analysis (HR 1.23, 95% CI, 0.45-3.40; *p* = 0.690) (Figure 3). There was no significant interaction by multiorgan transplantation status (p*interaction = 0.515), and the risk estimate was similar in individuals undergoing heart transplantation only (HR 1.22, 95% CI 0.38-3.88).

## Discussion

In this analysis comparing intermediate-term follow-up of CAV outcomes in HTx recipients of HCV NAT− and HCV NAT+ donor hearts, we found no significant difference in risk of CAV or year-over-year progression of CAV based on IVUS. Furthermore, there was no difference in risk for the composite outcome of ACS, PCI, or death over 6 years of follow-up by HCV donor status. These findings suggest that prior concerns of accelerated CAV and adverse outcomes with the use of HCV+ donor hearts are not present in the modern era of DAA therapy for HCV.

Our study advances prior research evaluating outcomes of HTx recipients from HCV-positive donors in the era of DAA therapies by using IVUS, a more sensitive assessment of CAV burden, over intermediate-term follow-up. Contemporary studies in the era of DAA therapies, which have often utilized traditional coronary angiography to define the presence and degree of CAV, found no association between donor HCV status and the development or progression of CAV after adjusting for known risk factors.^10^ Notably, many early studies had fairly limited follow-up periods post-transplantation or only assessed for prevalent CAV at one year post-transplantation. Like these studies, we did not find an association between HCV donor heart status and development or progression of CAV, nor in a composite outcome of ACS, PCI, or death, but extended these findings over a median follow-up of 6.6 years, with some recipients followed for nearly a decade. Importantly, we utilized IVUS rather than angiography, as IVUS has been shown to detect early, angiographically silent intimal thickening, providing a more sensitive measure of assessing CAV.^13^, ^14^

Furthermore, MIT is prognostically significant for future CAV events, with the development of an MIT ≥ 0.5 mm within the first year after transplant associated with higher rates of mortality, nonfatal major adverse cardiac events, and the development of angiographically confirmed CAV.^19^, ^20^ This becomes more pronounced with increasing degrees of MIT, as more severe intimal thickening is associated with a higher risk of clinically significant disease and adverse outcomes.^6^, ^14^, ^19^, ^21^, ^22^, ^23^ We assessed CAV using both the widely accepted MIT threshold of ≥0.5 mm as well as a more restrictive ≥ 0.7 mm cutoff, and HCV status was not associated with the development of CAV regardless of the cutoff evaluated. Although our overall rates of CAV development are high, the degree of intimal thickening is comparable to a study by Kim et al, which reported an average baseline MIT of >0.6 mm in over 50% of their cohort, as measured by a 6-week IVUS study.^24^ These findings confirm those of studies that showed no significant difference in the presence or severity of CAV, as defined by intimal thickness, between recipients of HCV NAT+ and HCV NAT− donor hearts, and further allays concerns about accelerated CAV in HCV donor hearts in the modern era of DAA therapy.

Year-over-year progression of intimal thickening is a well-established indicator of CAV severity and is associated with adverse outcomes, including re-transplantation, coronary revascularization, ACS, and mortality.^16^, ^19^, ^23^, ^24^, ^25^, ^26^ In our study, we observed no significant difference in the rate of MIT progression between recipients of NAT+ and NAT− donor hearts, consistent with prior research, but over a longer follow-up period.^27^ Additionally, we found no difference in the composite outcome of ACS, PCI, and all-cause mortality between the two groups. Importantly, in our study, all recipients of HCV viremic hearts (100%) achieved SVR within 12 weeks of treatment, suggesting that early viral clearance likely mitigates the inflammation and endothelial dysfunction associated with HCV and thereby reduces the risk for CAV.

Our findings reinforce the safety of transplanting HCV NAT+ donor hearts, showing no significant difference in outcomes over the intermediate term. However, CAV is a chronic and progressive process; therefore, longer-term follow-up is necessary to further characterize the risks in these populations. The treatment of hepatitis C is an evolving landscape, with many institutions, including our own, now transitioning to prophylactic DAA treatment. Peri-transplantation DAA therapies that completely prevent the development of viremia are likely to further mitigate any HCV-associated risks for CAV and adverse outcomes. Even with this pivot in HCV treatment peri-transplant, our findings are still relevant, as they represent a higher-risk population for the development of CAV due to the development of HCV viremia. Thus, the lack of any difference in CAV and outcomes between HCV NAT+ and NAT− recipients further reduces concerns about HCV donors in both the early and contemporary eras of DAA therapy for HCV donor organs. However, future studies are still needed to evaluate whether these associations persist over long-term follow-up.

### Limitations

Our study has important limitations. As a single-center study, our donor and recipient populations, as well as post-transplant care protocols, may not be generalizable to other centers. Additionally, the HCV-viremic cohort was relatively small, with only 22 recipients of HCV NAT+ donor hearts; thus, we may have lacked power to detect significant differences in CAV outcomes between groups, increasing the risk of Type II error. While our median follow-up was 6.6 years, CAV is a chronic process that often progresses beyond 5 years post-transplant, and longer-term follow-up may be necessary to detect differences in CAV risk. However, we used a more sensitive measure for identifying CAV with IVUS, and we suspect it is unlikely that substantial differences would develop later than our study period. Although IVUS is more sensitive for detecting early CAV, not all patients underwent serial coronary angiography at standardized timepoints, which limits the completeness of longitudinal CAV surveillance. However, our composite outcome, which does not rely on invasive testing, may be more practical and clinically meaningful.

Because early IVUS (performed within the first 3 months post-transplant) was not routinely performed at our institution, our ability to assess early donor-derived atherosclerotic burden is limited. Nonetheless, our use of modeling to evaluate changes in MIT over time improves the ability to assess for distinct progression of disease over time, regardless of baseline donor disease.

Additionally, since the onset of this study, further evidence has led to greater utilization of organs from DCD, rather than traditional donation after brain death. At the time of this study, our institution was infrequently performing DCD transplantation, and none of the individuals in this cohort underwent DCD; thus, our ability to compare transplantation of HCV-viremic hearts between recipients of DCD vs. donation after brain death organs is limited.

## Conclusions

With the advent of DAA therapies for the treatment of HCV, transplantation of HCV-positive donor hearts into HCV-negative recipients has become a standard practice to expand the donor pool. Though prior data from the pre-DAA era found an association between HCV+ donors and rapid development of CAV in transplant recipients, with the current widespread use of DAA therapy, this risk appears to be mitigated, though longer-term follow-up is still needed.

## Disclosure statement

The authors declare that they have no known competing financial interests or personal relationships that could have appeared to influence the work reported in this paper.

## Financial support

This research did not receive any specific grant from funding agencies in the public, commercial, or not-for-profit sectors.

## Declaration of Generative AI and AI-Assisted Technologies in the Writing Process

The content and original drafting of this manuscript were solely done by the authors. Following the initial draft, generative AI/AI-assisted technology (ChatGPT, Open AI) was used for grammatical editing and to enhance readability of language throughout the manuscript. After using this tool, the authors reviewed and edited the content as needed and take full responsibility for the content of the published article.

## References

[1] Colvin M.M., Smith J.M., Ahn Y.S. (2024). OPTN/SRTR 2022 annual data report: heart. Am J Transpl.

[2] Birs A.S., Kadosh B., Flattery E. (2022). Cardiac allograft vasculopathy in heart transplant recipients from hepatitis C viremic donors, data from two large academic transplant centers. J Heart Lung Transpl.

[3] Khush K.K., Cherikh W.S., Chambers D.C. (2019). The International Thoracic Organ Transplant Registry of the International Society for heart and lung transplantation: thirty-sixth adult heart transplantation report-2019; Focus theme: donor and recipient size match. J Heart Lung Transpl.

[4] Gonzalez S.A., Trotter J.F. (2018). The rise of the opioid epidemic and hepatitis C-positive organs: a new era in liver transplantation. Hepatology.

[5] Moayedi Y., Fan C.P.S., Gulamhusein A.F. (2018). Current use of hearts from hepatitis C viremic donors. Circ Heart Fail.

[6] Birs A.S., Bui Q.M., Gernhofer Y. (2024). Cardiac allograft vasculopathy outcomes among donation after circulatory death heart transplant recipients. JHLT Open.

[7] Kadosh B.S., Birs A.S., Flattery E. (2024). Cardiac allograft vasculopathy in heart transplant recipients from hepatitis C viremic donors. Clin Transpl.

[8] Haji S.A., Starling R.C., Avery R.K. (2004). Donor hepatitis-C seropositivity is an independent risk factor for the development of accelerated coronary vasculopathy and predicts outcome after cardiac transplantation. J Heart Lung Transpl.

[9] Woolley A.E., Singh S.K., Goldberg H.J. (2019). Heart and lung transplants from HCV-infected donors to uninfected recipients. N Engl J Med.

[10] Schlendorf K.H., Zalawadiya S., Shah A.S. (2018). Early outcomes using hepatitis C-positive donors for cardiac transplantation in the era of effective direct-acting anti-viral therapies. J Heart Lung Transpl.

[11] McLean R.C., Reese P.P., Acker M. (2019). Transplanting hepatitis C virus-infected hearts into uninfected recipients: a single-arm trial. Am J Transpl.

[12] Ramirez-Sanchez C., Kozuch J., Shah M.M. (2022). A pilot trial for prevention of hepatitis C virus transmission from donor to organ transplant recipient with short-course glecaprevir/pibrentasvir. Open Forum Infect Dis.

[13] Mendiz O.A., Gamboa P., Renedo M.F., Lev G.A., Favaloro L.E., Bertolotti A.M. (2021). Intravascular ultrasound for cardiac allograft vasculopathy detection. Clin Transpl.

[14] St Goar F.G., Pinto F.J., Alderman E.L. (1992). Intracoronary ultrasound in cardiac transplant recipients. In vivo evidence of "angiographically silent" intimal thickening. Circulation.

[15] Zakliczynski M., Swierad M., Zakliczynska H., Maruszewski M., Buszman P., Zembala M. (2005). Usefulness of stanford scale of intimal hyperplasia assessed by intravascular ultrasound to predict time of onset and severity of cardiac allograft vasculopathy. Transpl Proc.

[16] Seguchi O., Azarbal B., Mirocha J. (2023). Change in first-year intravascular ultrasound results predicts adverse events in heart transplant recipients: implications for clinical trial endpoints. Transplantation.

[17] Fine J.P., Gray R.J. (1999). A proportional hazards model for the subdistribution of a competing risk. J Am Stat Assoc.

[18] Shahandeh N., Kim J.S., Klomhaus A.M. (2024). Comparison of cardiac allograft vasculopathy incidence between simultaneous multiorgan and isolated heart transplant recipients in the United States. J Heart Lung Transpl.

[19] Kobashigawa J.A., Tobis J.M., Starling R.C. (2005). Multicenter intravascular ultrasound validation study among heart transplant recipients: outcomes after five years. J Am Coll Cardiol.

[20] Nelson L.M., Rossing K., Ihlemann N., Boesgaard S., Engstrøm T., Gustafsson F. (2020). Intravascular ultrasound-guided selection for early noninvasive cardiac allograft vasculopathy screening in heart transplant recipients. Clin Transpl.

[21] Skorić B., Čikeš M., Ljubas Maček J. (2014). Cardiac allograft vasculopathy: diagnosis, therapy, and prognosis. Croat Med J.

[22] Mehra M.R., Ventura H.O., Stapleton D.D., Smart F.W., Collins T.C., Ramee S.R. (1995). Presence of severe intimal thickening by intravascular ultrasonography predicts cardiac events in cardiac allograft vasculopathy. J Heart Lung Transpl.

[23] Costanzo M.R., Naftel D.C., Pritzker M.R. (1998). Heart transplant coronary artery disease detected by coronary angiography: a multiinstitutional study of preoperative donor and recipient risk factors. Cardiac Transplant Research Database. J Heart Lung Transpl.

[24] Kim I.C., Starling R.C., Khush K. (2024). Ten-year follow-up cohort of the everolimus versus azathioprine multinational prospective study focusing on intravascular ultrasound findings. J Heart Lung Transpl.

[25] Potena L., Masetti M., Sabatino M. (2015). Interplay of coronary angiography and intravascular ultrasound in predicting long-term outcomes after heart transplantation. J Heart Lung Transpl.

[26] Tuzcu E.M., Kapadia S.R., Sachar R. (2005). Intravascular ultrasound evidence of angiographically silent progression in coronary atherosclerosis predicts long-term morbidity and mortality after cardiac transplantation. J Am Coll Cardiol.

[27] Amancherla K., Feurer I.D., Rega S.A. (2024). Early assessment of cardiac allograft vasculopathy risk among recipients of hepatitis C virus-infected donors in the current era. J Card Fail.

