# Supplementary material for: Intermediate-term risk of cardiac allograft vasculopathy following heart transplantation from hepatitis C viremic donors in the era of direct-acting antiviral therapy
Source: JHLT Open. 2025 Oct 25;11:100416. doi: 10.1016/j.jhlto.2025.100416 (PMC12651725; doi:10.1016/j.jhlto.2025.100416)
Supplement: Supplementary file 1 — Supplementary material [file mmc1.docx]

**Supplemental Table 1: Angiographic Data Year 1**

|  | NAT Negative (N=109) | NAT Positive (N=22) | p-value |
| --- | --- | --- | --- |
| ISHLT Grade, N (%) |  |  | 0.248 |
| Grade 0 | 83 (79.1) | 17(81.0) | >0.999 |
| Grade 1 | 19 (18.1) | 2 (9.5) | 0.523 |
| Grade 2 | 2 (1.9) | 1(4.8) | 0.424 |
| Grade 3 | 1 (1.0) | 1 (4.8) | 0.307 |
| Average MLA (mm^2^), mean (SD) |  |  |  |
| MLA Left Main | 17.8 (6.7) | 16.0 (5.9) | 0.611 |
| MLA pLAD | 11.7 (3.9) | 10.6 (3.3) | 0.505 |
| MLA mLAD | 7.5 (2.7) | 10.7 (5.3) | 0.235 |
| MLA dLAD | 4.1 (0.9) | - | - |
| Location of MIT, N (%) |  |  | 0.523 |
| Left Main | 25(25.5) | 8 (38.1) | 0.285 |
| pLAD | 50 (51.0) | 11 (52.4) | >0.999 |
| mLAD | 21 (21.4) | 2 (9.5) | 0.360 |
| dLAD | 2 (0.2) | 0 (0) | >0.999 |
